# The Association between Education Outside the Classroom and Physical Activity: Differences Attributable to the Type of Space?

**DOI:** 10.3390/children8060486

**Published:** 2021-06-07

**Authors:** Mads Bølling, Erik Mygind, Lærke Mygind, Peter Bentsen, Peter Elsborg

**Affiliations:** 1 Health Promotion Research, Steno Diabetes Center Copenhagen, The Capital Region of Denmark, Niels Steensens Vej 6, DK-2820 Gentofte, Denmark; peter.elsborg@regionh.dk; 2 Department of Geosciences and Natural Resource Management, University of Copenhagen, Rolighedsvej 23, DK-1958 Frederiksberg C, Denmark; emygind@ign.ku.dk (E.M.); peter.bentsen@regionh.dk (P.B.); 3 Center for Clinical Research and Prevention, Copenhagen University Hospital—Bispebjerg and Frederiksberg, The Capital Region of Denmark, DK-2000 Frederiksberg, Denmark; laerke.mygind.groenfeldt@regionh.dk; 4 School of Psychology, Cognitive Neuroscience Unit, Deakin University, Waurn Ponds, VIC 3216, Australia; 5 Department of Public Health, Unit of Medical Psychology, University of Copenhagen, DK-1014 Copenhagen, Denmark

**Keywords:** learning environment, green space, learning outside the classroom, movement behaviour, movement integration, outdoor learning, physical activity, udeskole

## Abstract

Education outside the classroom (EOtC) has become an attractive approach, not only for learning but also for health. This explorative, cross-sectional study investigated children’s sedentary behaviours (SED), light physical activity (LPA) and moderate-to-vigorous PA (MVPA) on school days with an EOtC session in green space compared to school days with EOtC in other environments and without EOtC. Teachers from 17 Danish school classes practised EOtC for one school year on a weekly basis and self-reported the characteristics of the EOtC environment. The pupils’ PA was device-measured for seven consecutive days in a random period during the school year with AX3 accelerometers. Across 617 pupils (age 9–13 years), PA intensity cases (*N* = 2264) on school days (8:10–14:00 h) with (*n* = 317) or without (*n* = 1947) EOtC were included in a mixed-effects regression analysis. Mean exposure to EOtC was 262 min per session. School days with green EOtC (e.g., parks, forests and nature schools) were associated with (mean, [95% CI]) −24.3 [−41.8, −7.7] min SED and +21.3 [7.7, 36.4] min LPA compared to school days with non-green EOtC (e.g., cultural and societal institutions or companies) and with +6.2 [−0.11, 11.48] min MVPA compared to school days with a school-ground EOtC. No sex differences were found. In conclusion, school days with green EOtC must be considered promising to counteract children’s sedentary behaviours during school hours.

## 1. Introduction

Physical inactivity is a concern in most Western and in high-income countries [1]. Children’s daily physical activity (PA) has become increasingly polarised over the last 20 years. Difference between highly active and inactive children has increased and an increasing number of children are not meeting the recommended PA guidelines [2,3]. Many different efforts have been made to increase children’s PA and create solutions to change this problematic development [4]. A central context in which these sedentary lifestyle challenges should be tackled is schools [5]. Across the week, the school is the context where children spend most time on average, only succeeded by their home [6,7].

In addition to encouragement to walk and cycle, e.g., to and from school [8], PA-increasing initiatives during school hours may involve extracurricular time allocated to movement activities or delivery of the curriculum content by the teacher with an explicit focus on integrating movement [9,10]. However, such extracurricular approaches require additional resources and may be perceived as being outside the primary goals and concerns of teachers [11]. By contrast, movement implicitly integrated with primary pedagogical and didactical purposes, i.e., an add-in approach, might yield more acceptance with teachers and therefore increase the implementation potential [12,13].

### 1.1. Use of Green Space Outside the Classroom

Several studies link green space to increased PA and suggest that such locations are particularly popular amongst children and youth. For instance, a study from schools in New Zealand showed that the proportion of time young adolescents spent in green space is associated with more time spent in MVPA [14]. School ground greening is a widely used approach to stimulate academic learning activities [15]. If the school grounds are integrated in the teaching lessons, they might offer opportunities for different types of active play and learning that stimulate PA among pupils [16]. School grounds with a high level of natural features and a diverse landscape seem to, in particular, stimulate PA [17,18,19]. However, beyond the school ground, too, there are excellent opportunities for learning but potentially also for increasing movement. Such a teaching approach is described as education outside the classroom (EOtC). In this approach, pupils are taught traditional school topics outside the school buildings or school grounds in, for example, nature, culture and societal institutions, or companies [20]. If EOtC is practiced regularly, it is often referred to as the Scandinavian concept of ‘udeskole’ (literally meaning outside schooling) [20,21,22]. EOtC is characterised by collaborative, action-centred, experiential, problem-based and thematic learning processes that involve teaching and learning activities outside the school buildings and that align with teachers’ curricular obligations [20].

Pupils value relocation of some school day teaching hours to places outside the school ground so that they can access green space for possibilities to be more physically active [23]. This seems to be especially favourable for boys [18,24,25,26,27]. Often, EOtC includes learning activities that demand pupils to move, e.g., measuring and estimating the volume of trees in maths class or incorporating tag-and-relay races in language lessons [12]. In April 2020, EOtC gained increased attention worldwide as a strategy to reduce the spread of infection when reopening schools during the COVID-19 pandemic [28]. When schools reopened, many were obligated to teach outdoors. Teacher gained experiences using EOtC and more regular physical practice during curriculum-based teaching became an option.

Few studies have analysed the association between EOtC and pupils’ PA [29]. A cross-sectional study on pupils in Grades Three to Six (mean age 9–12 years) showed that EOtC has a positive impact on overall weekly moderate-to-vigorous PA (MVPA) for boys [27]. For both sexes, light-intensity PA (LPA) is higher on school days with EOtC compared to normal school days with or without a physical education (PE) lesson [30]. In a case study in lower secondary school with pupils in Grades Four to Six, one of four classes was taught using EOtC in green space [25]. School days with EOtC conducted in green space were associated with higher PA levels compared to standard school days without PE. No difference was found between school days with EOtC at cultural institutions compared to the standard school days. Mean PA levels among boys were significantly higher than among girls in all measured school day settings except on normal school days with a PE lesson [25]. In addition, weekly teaching in forests in lower secondary school yields PA levels significantly higher compared to other school days [26,31]. Regular-practised EOtC simultaneously with positive PA benefits also has an effect on school core outcomes [29], i.e., school motivation [32], social well-being [33] and learning [34].

The above-mentioned research indicates that EOtC is a profitable approach to increasing pupils’ time spent in high and low PA intensities, perhaps moderated by school days with EOtC in green space. Compared with boys, girls seem to have a more questionable PA benefit. However, there is a lack of studies exploring the association between EOtC conducted in green space and pupils’ PA compared to other EOtC environments.

### 1.2. Research Aims

The objective of this explorative, cross-sectional study was to investigate PA levels on school days with an EOtC session in green space compared with other school days settings, i.e., settings with an EOtC session in other types of environments and school days without any EOtC. Aim One was to study sedentary behaviour (SED) comparing school days with an EOtC session in green space with the other school days settings. Aim Two was to study light-intensity PA (LPA) comparing school days with an EOtC session in green space with the other school days settings. Aim Three was to study moderate-to-vigorous PA (MVPA) comparing school days with an EOtC session in green space with the other school days settings. Aim Four was to study gender differences for each of the PA levels, i.e., SED, LPA, MVPA, when making the comparisons.

## 2. Materials and Methods

### 2.1. Setting

The present study was part of the larger quasi-experimental TEACHOUT study set in schools across Denmark [35]. In Denmark, regular EOtC has been adopted by teachers in at least 19.5% of all general schools in the country [36]. Green space is the preferred environment among teachers using EOtC on a regular basis [37]. In Denmark, EOtC is not mandatory, but in 2014, the Ministry of Education launched a new school reform that included opportunities for using various EOtC-friendly teaching approaches [38]. In addition, the reform included a mandatory 45 min of PA per day on top of the weekly PE lesson that is restricted to one double lesson lasting about 60 min.

### 2.2. Recruitment

Recruitment of school classes was done by contacting municipalities and schools known to practise EOtC regularly, i.e., based on contact information from a national survey and through professional networks within the research group [30,35]. Classes were recruited to evaluate the TEACHOUT EOtC intervention by investigating the association between EOtC and pupils’ PA and the effect on academic learning, motivation for learning, social relations and well-being among the same group of school-aged pupils from Grades Three to Six (mean age 9–12 years) [35].

Recruitment was based on the class teachers’ and school management’s willingness to implement EOtC on a regular basis for one school year. To compare parallel classes, teachers from the same grade level from each school were predominately enrolled in pairs: one class teacher agreed to use EOtC as part of their curriculum-based teaching in one class, while the other parallel class teacher agreed to maintain teaching as usual with an expected minimal use of EOtC. The recruited schools were located in both rural and urban areas.

### 2.3. The TEACHOUT Intervention

The TEACHOUT intervention included a two-day teacher-training course on the pedagogy of EOtC, followed by illustrative examples of using this practice in various school subjects. Intervention and control classes both received information about study participation [39]. Thereafter, teachers were to implement EOtC on a regular basis over the course of one school year (August 2014 until the end of May 2015), a minimum of 300 min per week, divided into one to two weekly sessions, including preparation, transportation, breaks and evaluation in the classroom. A school day with an EOtC session was defined as 45 min or more of continuous curriculum-based teaching outside the school buildings. The teachers could decide which subjects they wanted to teach and in which environments outside the classroom.

### 2.4. Sampling of PA Cases with or without Different Types of EOtC Sessions

The study cohort was device-based monitored cases of PA intensities drawn from a sample of 617 pupils (341 girls, 276 boys) with a mean age of 10.9 years (range 8.7–13.6 years) across 37 classes (9 classes from Grade Three with 135 pupils, 13 classes from Grade Four with 227 pupils, 9 classes from Grade five with 156 pupils and 6 classes from Grade Six with 99 pupils) from 15 schools in the TEACHOUT study. Pupils with seven consecutive full days of accelerometer wear time and complete data on time spent in activity domains during school hours (e.g., recess, PE, classroom teaching and EOtC) were included in the study. Each pupil with at least *n* = 1 monitored PA case with or without an EOtC session (mean ± SD, 3.67 ± 1.48 PA cases per pupil) was included. A PA case was defined as PA intensity data from one school day and from one pupil. To be sure to only capture school day PA activity, 8:10–14:00 h school operation time (350 min) was applied. Up to five cases were drawn from each of the 617 pupils. Of these pupils, 76 had *n* = 1, 100 had *n* = 2, 58 had *n* = 3, 101 had *n* = 4 and 282 had *n* = 5 cases of PA. In total, the study sample had *N* = 2264 cases with or without an EOtC session (see Supplementary Materials, Table S1). A total of 166 pupils had at least *n* = 1 PA case (mean ± SD, 1.11 ± 0.32 cases per pupil) on school days, which included EOtC in green space. Of these 166 pupils, 104 had at least *n* = 1 other PA case (mean ± SD, 3.13 ± 1.32 cases per pupil) on school days with or without a different type of EOtC session.

### 2.5. Data Collection and Measurements

Data were collected between November and June of the 2014–2015 school year, where classes were visited at their respective schools. Each participating child reported their birth date and had their height (Leicester height measure) and weight (OMRON BF212 body composition monitor) measured [30,40].

#### 2.5.1. Physical Activity

PA was measured continuously day and night over 10 days with Axivity AX3 accelerometers (Axivity, Newcastle, UK), using a 15-s epoch length for data transformation [30]. Information about the validity and feasibility of the AX3 accelerometer is described in [40,41]. The AX3 accelerometer can be attached to the skin on the loins and thighs with a self-adhesive plastic cover and thus enables measurements around the clock. In addition, it is waterproof, and it has a temperature sensor that can be used to estimate wear time [40,41]. For detailed information about data collection, PA level cut points, specific methods, and the validity and compliance of accelerometer-derived measurements of PA in the TEACHOUT study, see [30,40].

#### 2.5.2. Child Diary Report of PA Periods and School Day Activities

In each participating class, three pupils had the responsibility to fill in a class diary during the period of PA measurements and were asked to provide diary information about school day activity domains [30].

#### 2.5.3. Teacher Report of Setting and Time on Different Types of Traditional School Days and Days with EOtC

Using a online EOtC-monitoring tool, teachers reported where each EOtC session took place: in green space (e.g., park, forest, field, beach or lake), at a nature school, at a cultural and societal institution (e.g., museum, cemetery or library), at companies (e.g., store, factory or technical facility) and in the school grounds outside the school buildings. For each EOtC session, the teachers also reported whether the session was conducted outdoors, indoors or both out- and indoors. In addition, teachers reported a short qualitative description of the environment used in each EOtC session. The monitoring was completed on daily basis by teachers logging to a secured website making their report in a survey. Finally, data were classified into three EOtC environment categories: green EOtC, school days with EOtC sessions conducted in parks, forests or nature schools; non-green EOtC, school days with EOtC sessions conducted in cultural and societal institutions or companies; and school-ground EOtC, school days with EOtC sessions in the school’s outdoor areas. The categorisation was based on all available information from each EOtC session (see Supplementary Materials, Table S1). Three of the authors made an individual categorisation of 17 different EOtC sessions, which were then compared. The overall agreement was 92%. On four occasions, 100% agreement was not demonstrated. The discrepancy was discussed, and an agreement of the final categorisation was established.

Reports of the time spent in EOtC sessions were used to cross-check class diary information. The mean exposure to EOtC was 262 (range 90 to 640) min per session (average across EOtC sessions; see Supplementary Materials, Table S1). A school day with a PE lesson was defined as a school day with 45 min or more of PE.

Teachers recorded the duration of each reported EOtC session, the duration of break/free time, transportation and the mode used in conjunction with the session. Mode could be recorded as either active (e.g., walking or cycling), passive (e.g., bus, train, metro or car) or both. Subsequently, data on transportation were transformed to two continuous variables accounting for, respectively, active and passive mode duration (min).

Using the class diaries, class timetables and data from the online EOtC-monitoring tool, cases of PA intensities were sorted into five school day setting categories used in the statistical analyses (see Table 1): school days with green EOtC without a PE lesson (default); non-green EOtC without a PE lesson; school days with an EOtC session in the school grounds and without a PE lesson (school-ground EOtC); a traditional school day without a PE lesson or an EOtC session (without PE or EOtC); and a traditional school day with a PE lesson (PE without EOtC).

### 2.6. Statistical Analyses

One linear-mixed model analysis was performed for each of the three main outcomes: SED, LPA and MVPA. Fixed effects were the five-category school day setting variable. Cases nested in pupils, in classes and in schools were included as random effects. In each of the three models’ (one model for each PA intensity: SED, LPA and MVPA) intensities were adjusted for duration of EOtC session (two cases exceeded an 350 min exposure to EOtC), duration of active and passive transportation during the EOtC session, duration of break/free time during the EOtC session, sex, age and BMI. Post hoc estimations of marginal means were conducted.

To investigate sex differences across all school day settings simultaneously, a continuous ‘school day setting × sex’ interaction term was added to each of the three models. Sex differences were investigated by comparing green EOtC to each of the four other school day settings, separately adding a categorical ‘school day setting × sex’ interaction term to the models. In the case of a statistically significant difference between sexes, *p*-values were calculated for each sex. Statistical analyses were performed using the lme4 [42] and lmerTest [43] packages in R version 1.2.5042 [44]. Post hoc estimations of marginal means were performed using the emmeans package for R [45]. The R function ‘confint’ were used for 95% confidence interval estimation. The significance test was two-tailed, and *p* < 0.05 was considered statistically significant.

## 3. Results

**Figure 1:**
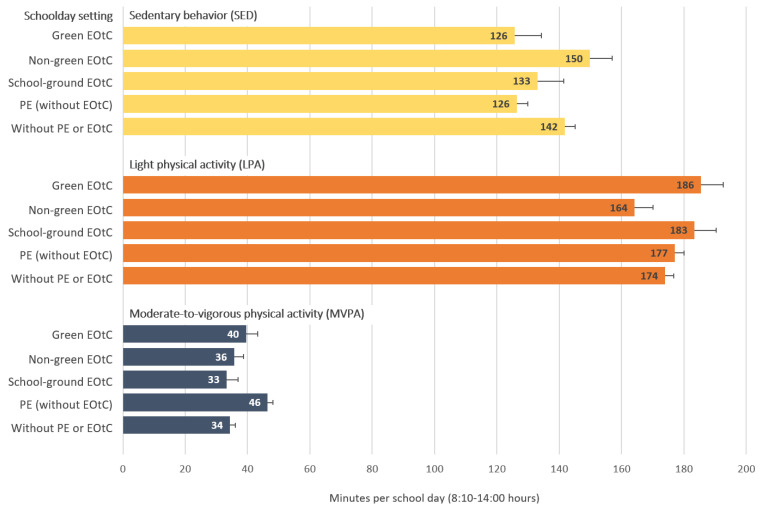
Minutes spent in SED, LPA and MVPA during the school day in different school day setting categories. Estimated marginal means of the linear-mixed models with SED, LPA and MVPA as fixed effects separately with cases nested in pupils, in classes, and in schools as random effects. Models are adjusted for duration of EOtC sessions, duration of active and passive transportation and duration of break/free time during EOtC sessions; sex; age; and BMI. Error bares represent the ±SD of its sampling distribution.

Figure 1 shows the time spent in SED, LPA and MVPA in the five different school day settings. An overview of findings is presented in Table 1 (see full results of the mixed models in Supplementary Materials, Tables S2–S10).

### 3.1. Aim One: Sedentary Behaviour (SED)

Descriptive statistics (see Table 1) showed that the time spent in SED was higher in all the school day settings compared with school days with green EOtC (range 0.22% to 6.94%).

The time spent in SED on school days with green EOtC was 24.3 min less (*p* = 0.005; 95% CI = 7.7 min to 41.8 min) compared with school days with non-green EOtC. No difference was found compared with school days with school-ground EOtC (*p* = 0.316) and traditional school days with a PE lesson and without an EOtC session (*p* = 0.935). A tendency to a higher SED level was found on traditional school days without a PE lesson (*p* = 0.080) compared with school days with green EOtC.

### 3.2. Aim Two: Light-Intensity Physical Activity (LPA)

Descriptive statistics (see Table 1) show that the time spent in LPA was lower in all school day settings (range −6.08% to −0.59%) compared with school days with green EOtC.

The time spent in LPA on school days with green EOtC was 21.3 min more (*p* = 0.003; 95% CI = 7.7 min to 36.4 min) compared with school days with non-green EOtC. No difference was found compared with school days with school-ground EOtC (*p* = 0.731), school days with a PE lesson and without an EOtC session (*p* = 0.284) or traditional school days without a PE lesson or an EOtC session (*p* = 0.129).

### 3.3. Aim Three: Moderate-to-Vigorous Physical Activity (MVPA)

Descriptive statistics (see Table 1) show that green EOtC time spent in MVPA was slightly higher on school days with non-green EOtC, school days with school-ground EOtC and traditional school days without a PE lesson or an EOtC session (mean, −1.10% to −1.78%). On school days with a PE lesson, MVPA was higher (mean, 1.98%) compared with school days with green EOtC.

The time spent in MVPA on school days with green EOtC was 6.2 min more (*p* = 0.044; 95% CI = −0.11 min to 11.48 min) compared with school days with school-ground EOtC, while no difference was found compared with any of the other school day settings. A tendency to a higher MVPA level was found on school days with a PE lesson and without an EOtC session (*p* = 0.081) compared with school days with green EOtC. School days with non-green EOtC (*p* = 0.285) and traditional school days without a PE lesson or an EOtC session (*p* = 0.187) showed no statistically significant difference from school days with green EOtC.

### 3.4. Aim Four: Sex Differences

Comparing school days with green EOtC with each of the four other school day settings showed no sex differences (see Table 1). However, comparison between school days with green EOtC and school days with a PE lesson showed a borderline significant difference in the time spent in LPA (*p* = 0.073). Boys spent less time in LPA on school days with a PE lesson (mean −19.5 min, 95% CI = −46.8 min to −1.8 min) compared with girls (mean −3.9 min, 95% CI = −23.9 min to 15.1 min). Individual differences between school days with green EOtC and school days with a PE lesson were non-significant for girls (*p* = 0.693) and boys (*p* = 0.091).

## 4. Discussion

When relocating teaching to places outside the school cadastral, our findings call for EOtC to be conducted in green space when the aim is to promote more PA. Although EOtC conducted in green space is associated with a modest time spent in MVPA, i.e., 6.2 min (95% CI = −0.11 min to 11.48 min) compared with school days with school-ground EOtC, school days with an EOtC session in green space seem equally beneficial compared with school days with an EOtC session in the school grounds in terms of light-intensity PA. Both settings seem to allow pupils to move during teaching outside the school buildings, where the teacher’s focus is on the academic context and learning process and not in particular on physical activity.

This explorative, cross-sectional study is the first to investigate PA levels at different intensities (SED, LPA and MVPA) on school days with three types of EOtC sessions. In total, 185 PA intensity cases (i.e., PA intensity data from one school day drawn from one pupil) collected across ten EOtC sessions in green space were compared to 59 PA intensity cases from three school days with EOtC sessions in non-green space (e.g., art museum, theatre and grocery store) and 73 cases from four school days with an EOtC session in the school grounds. Further, comparisons of school days with green EOtC were made with 521 and 1426 cases from traditional school days, respectively, with or without a PE lesson.

The results show that school days with an EOtC session in green space provide less SED and more LPA compared with other school days with an EOtC session outside the school ground. All the results are adjusted for influence by the length of the session and the amount of time used for breaks and active or passive transportation. Although there was a tendency of less SED time on school days with a green EOtC session compared with traditional school days (mean 16.3 min, 95% CI = −1.5 min to 35.3 min, *p* = 0.080; see Table 1), no other difference in the time spent in SED or LPA was found compared to school days with an EOtC session in the school grounds, school days with a PE lesson without an EOtC session or traditional school days. These findings are not surprising as pupils in both green EOtC and school-ground EOtC school day settings are taught outside the classroom and as PE lessons on traditional school days are expected to reduce SED activity and increase PA. Further, school days with an EOtC session in the school grounds were associated with a lower MVPA level compared with school days with an EOtC session in green space. In addition, we found a tendency to a higher MVPA level on traditional school days with a PE lesson and without an EOtC session compared with school days with an EOtC session in green space (mean ± SD 6.9 min, 95% CI = −0.2 min to 17.2 min, *p* = 0.081; see Table 1).

A number of studies support the findings of increased LPA and MVPA through regular teaching outside the school buildings [16,25,26,31]. If the school grounds are characterised by a non-uniform environment with trees, shrubs and boulders, the effect could be larger in the light-to-moderate-intensity PA range, as reported by school personnel [18], which could explain lower MVPA levels found in our study. The physical environment in the school (indoor and outdoor) may afford different activity patterns [24]. The schools in the study were located in both rural and urban areas; however, no data on the schools’ local physical environment were collected. Green space around the school might be planned differently and have different conditions to evolve, e.g., the difference between a city park, a forest and a thicket.

### 4.1. Sex-Independent Benefit of EOtC in Green Space

None of the findings differed significantly between sexes. This sex-independent benefit we found seems reasonable, as although PA affordances during EOtC is most mentioned by boys, interviews with both sexes showed that teaching lessons in green space allow pupils to be physically active [24]. However, our finding confutes previous findings that have highlighted sex dependency in favour of boys spending more time in MVPA during school weeks with an EOtC session [27] and boys being more active during EOtC sessions conducted in green space [25]. However, school days with an EOtC session are positively associated with more LPA for both sexes compared with other school days with or without PE lessons [30]. Still, the sex independence may vary across age. The pupils in this study were analysed as a homogeneous group: pupils from Grades Three to Six. In general, activity levels decrease with age and vary across different age groups [8]. Also, boys have a higher level of physical activity compared with girls, and we can speculate whether pupils from different sex-dependent age groups react differently to the movement afforded during EOtC. EOtC might be planned differently by teachers across grade levels and in respect to the scope of the lesson, unintentionally providing different movement options [13], e.g., play and games with younger pupils.

Giving the dependent nature between SED, LPA and MVPA, descriptive statistics highlight that the extended time spent by both sexes in MVPA on school days with an EOtC session in green space is primarily replaced with less time spent in SED compared with school days with school-ground EOtC. From a public and population health perspective, this is an interesting finding. Our findings show that compared with traditional school days with a PE lesson, school days with an EOtC session seem to increase LPA of pupils in favour of boys. Although this sex-dependent finding is only borderline significant (*p* = 0.073), boys, compared with girls, spent more time in LPA on school days with green EOtC compared with school days with a PE lesson. Although a non-significant finding (*p* = 0.091), this study shows that EOtC conducted in green space might be able to compete with the otherwise PA-promoting PE lessons, reducing boys’ sedentary behaviour. This hypothesis calls for re-investigation with a larger sample.

### 4.2. The Benefit of LPA above MVPA

Although our results showed that EOtC is beneficial for LPA and less SED, we found a more unclear picture of MVPA on school days with an EOtC session in green space. In terms of MVPA, our findings do not markedly refine the conclusion from previous studies [25,30]. School days with an EOtC session in green space only showed a higher, but minimal, MVPA level compared with school days with an EOtC session in the school grounds and only a borderline difference from school days with a PE lesson without an EOtC session. According to [25,30], EOtC is associated with more time spent weekly in MVPA but only among boys. When comparing these results, in previous studies, the movement benefit of transportation time is included in the measures. Our aim was to investigate the time spent in places outside the classroom, and therefore the statistical models were adjusted for an eventual benefit of transportation.

In terms of LPA, the health benefits seem evident, as demonstrated in a German intervention, where LPA in the outdoor teaching setting was strongly associated with a decline in pupils’ cortisol levels [46]. The benefit of LPA has gained increased attention through studies focusing on the adult population. In addition, LPA among young people may be a contributor to a reduction in lifestyle factors such as diabetes, obesity and mortality risk later in life. LPA is argued to be included as a specific measure in PA guidelines [47,48]. Implemented across the entire population [49], but not the least regularly in school, where all children spend a large part of their week, EOtC in green space is a promising add-in intervention with potential for increasing health [12].

In a school setting, the possibility to promote LPA on school days with an EOtC session in green space once every school week must be seen relative to other potential advantages of relocating teaching to green space for school health and learning. Green space is seen as a distinct source of inspiration in the curricular teaching, which, among other aspects, characterises the Scandinavian EOtC tradition [50]. Although there is no straightforward association between school greenness and academic performance [51], immersive nature activities (e.g., school-based teaching in green space) seem to promote pupils’ and adolescents’ self-esteem, self-efficacy, resilience and academic and cognitive performance [52], as well as learning motivation [32]. Above all, the benefit of EOtC must be seen in the scope of its add-in nature to promote PA and health. EOtC does not compromise the so-called core business of schools—academic learning and well-being—and is in line with general pedagogical and didactical purposes [13] compared to explicit movement integration strategies in lessons or school time [9].

### 4.3. Strengths and Limitation

This study is the first to investigate the association between school days with an EOtC session in green space and pupils’ PA, comparing various EOtC and non-EOtC school day settings. Although only based on data from 17 EOtC sessions, ten of which were in green space (see Supplementary Materials, Table S1), the study was based on a large sample of cases of PA intensities during school hours comprising EOtC or not, across 37 classes. Further, the nested structure of data (pupils in classes in schools) was accounted for in the analysis. Compared to other studies on the association between school time exposure to green space and pupils’ PA [17,18,19], this study investigated a teaching practice combining learning activities that to a higher extent stimulate pupils to move based on how teachers decide to use the environment, e.g., green space and other environments outside the school buildings or school grounds. However, the findings must be evaluated based on several limitations.

This non-randomised, cross-sectional study included a sample of cases of PA intensities drawn from pupils across the TEACHOUT study intervention and control groups who were included if EOtC at the class level was equal or more than 45 min a week [35].

The environments where EOtC sessions were conducted—embedded in one of the key study variables (i.e., school day setting)—were reported, mainly quantitively, by teachers and subsequently categorised by the researchers using an inter-researcher evaluation between three researchers. A combined GPS- and GIS-based method to assess the greenness of the environments used for the EOtC sessions would have been a more objective measure [53]. The non-documented difference between green EOtC and school-ground EOtC could be related to some kind of greenness in the school grounds.

The statistical models are adjusted for the eventual effect of the transportation associated with relocating teaching outside the school grounds, because the purpose of this study was to evaluate the effect of the widespread use of the EOtC environment, in particular green space. However, green space with the desired quality can involve transportation time, which might add further PA to the school days with an EOtC session [54]. Out of the ten EOtC sessions in green space, eight primarily involved active transportation (range 10–120 min), while two of the three EOtC sessions in non-green environments (i.e., a museum and a theatre) primarily involved passive transportation (i.e., 90 and 120 min). Although transportation could include active movement (e.g., walking or cycling), the aim of this study was solely to investigate the EOtC session teaching hours used in green space.

Other aspects not considered were the weather and the data collection taking place during the Northern Hemisphere winter. EOtC is characterised by teaching outside school buildings, school grounds (13 EOtC sessions investigated in this study; see Supplementary Materials, Table S1) and green space outside school grounds (10 EOtC sessions investigated in this study; see Supplementary Materials, Table S1). PA data were collected between November and May, and seasonal and daily weather conditions may have affected pupils’ activity levels [55].

The role of the teacher must also be considered. The learning activities applied during a school day and how much PA the learning activities afford may vary. Much of the difference between school day settings, and especially between the different environments used for EOtC, explain the diversity in PA. In addition to the teachers’ EOtC teaching experience [13], their motivation to use EOtC across each involved class could also affect the pupils’ motivation for the activities and how physically active they are [56]. The teacher’s intention with the specific lessons may also affect options for the pupils being physical active.

Based on its explorative nature and considering the above-mentioned limitations, the findings of this educational intervention study must be considered preliminary [57]. Future studies need to test, in randomised trials with a large sample of EOtC sessions, whether EOtC in green space has an advantage in terms of promoting both LPA and MVPA for both sexes compared to traditional school days without a PE lesson. Studies have demonstrated increased MVPA benefits of EOtC for boys [25,27], while the three EOtC environments explored in the current study find EOtC equally beneficial for both sexes.

## 5. Conclusions and Perspectives

This explorative, cross-sectional study shows that school days with an EOtC session in green space and without a PE lesson are associated with more LPA and less sedentary behaviour compared with school days with an EOtC session in cultural and societal institutions or companies and without a PE lesson. As no inferential statistical analysis show differences between sexes, school days with an EOtC session in green space seem beneficial to both girls’ and boys’ LPA. To what degree MVPA is stimulated compared to school days with an EOtC session conducted in the school grounds is more unclear. Teachers’ focus on academic learning in EOtC sessions might explain the less MVPA compared to other studies mentioned in this paper, but one explanation could be the specific focus on EOtC settings and sessions and not including active transport.

This study is the first to investigate the association between EOtC in green space and pupils’ PA, comparing school days with EOtC sessions in various environments and non-EOtC school day settings based on a large sample of PA intensity cases. However, the findings must be considered preliminary. Therefore, we recommend future hypothesis-driven investigations in order to replicate this study and ensure reliability of the findings.

Worldwide, reopening of schools during the COVID-19 pandemic meant increased use of outdoor environments in teaching lessons [28]. In Denmark, a reduction in virus transmission was the main argument for the relocation of lessons to outdoors [58]. Nevertheless, a side effect was the gained collective level of experience with teaching in outdoor environments and thus a chance for more regular practice of curriculum-based teaching in green space, which has the potential to simulate pupils’ cardio-vascular health and promising learning perspectives. Knowledge of the impact on learning and health must be a top priority to inform evidence-based upscaling for the continuation of the experience gained during school reopening.

## Figures and Tables

**Table 1:** Proportion of school days spent in SED, LPA and MVPA in different school day setting categories.

		Green EOtC (Default) ^a^	Non-Green EOtC	School-Ground EOtC	PE (without EOtC)	Without PE or EOtC
**Fixed effect**	***n* cases**	185	59	73	521	1426
**SED**	**All % (95% CI)**		6.94 (2.19, 11.95)	2.08 (−1.89, 6.28)	0.22 (−4.91, 5.71)	4.65 (−0.42, 10.09)
	***p***		0.005 **	0.316	0.935	0.080 *
	**Girls % (95% CI)**		6.97 (1.02, 13.05)	2.48 (−2.46, 7.55)	−2.08 (−8.63, 4.65)	3.36 (−3.13, 10.04)
	**Boys % (95% CI)**		7.74 (−0.00, 16.30)	3.21 (−3.02, 10.02)	4.58 (−3.24, 13.48)	7.53 (−0.17, 16.34)
	***p*^b^**		0.709	0.602	0.410	0.711
**LPA**	**All % (95% CI)**		−6.08 (−10.40, −2.20)	−0.59 (−4.61, 2.66)	−2.37 (−7.17, 1,85)	−3.32 (−8.07, 0.86)
	***p***		0.003 **	0.731	0.284	0.129
	**Girls % (95% CI)**		−7.26 (−12.42, −2.30)	−1.05 (−5.34, 3.08)	−1.12 (−6.84, 4.31)	−3.40 (−9.07, 1.98)
	**Boys % (95% CI)**		−5.07 (−12.39, 0.91)	−1.28 (−6.71, 3.48)	−5.56 (−13.36, 0.52) *^†^	−4.60 (−12.31, 1.39)
	***p*^b^**		0.398	0.810	0.073 *	0.835
**MVPA**	**All % (95% CI)**		−1.10 (−3.17, 0.83)	−1.78 (−3.28, 0.03)	1.98 (−0.07, 4.92)	−1.48 (−3.50, 0.81)
	***p***		0.285	0.044 **	0.081 *	0.187
	**Girls % (95% CI)**		0.17 (−1.88, 2.23)	−1.66 (−3.39, 0.09)	3.06 (0.77, 5.38)	−0.10 (−2.37, 2.20)
	**Boys % (95% CI)**		−3.96 (−7.99, 0.17)	−2.91 (−6.37, 0.56)	−0.34 (−4.47, 3.96)	−4.18 (−8.26, 0.05)
	***p*^b^**		0.303	0.391	0.160	0.672

*N* = 2264 cases. ^a^ Reference category. ^b^ ‘School day setting × sex’ interaction analysis. %, proportion of the school day (8:10–14:00 hours). * *p* < 0.1, ** *p* < 0.05. ^†^ Exact *p*-value = 0.091.

## Data Availability

Data is available on request with the authors.

## Supplementary Materials

The following are available online at https://www.mdpi.com/article/10.3390/children8060486/s1, Table S1: sample of devise-based measured PA cases and EOtC sessions. Table S2: SED full mixed model results. Table S3: LPA full mixed model results. Table S4: MVPA full mixed model results. Table S5: Girls’ SED full mixed model results. Table S6: Girls’ LPA full mixed model results. Table S7: Girls’ MVPA full mixed model results. Table S8: Boys’ SED full mixed model results. Table S9: Boys’ LPA full mixed model results. Table S10: Boys’ MVPA full mixed model results.

## References

[1] Ding D., Lawson K.D., Kolbe-Alexander T.L., Finkelstein E.A., Katzmarzyk P.T., Mechelen W.v., Pratt M. (2016). The Economic Burden of Physical Inactivity: A Global Analysis of Major Non-Communicable Diseases. Lancet.

[2] Dalene K.E., Kolle E., Northstone K., Møller N.C., Grøntved A., Wedderkopp N., Kriemler S., Page A.S., Puder J.J., Reilly J.J. (2020). Variations in Accelerometry Measured Physical Activity and Sedentary Time across Europe—Harmonized Analyses of 47,497 Children and Adolescents. Int. J. Behav. Nutr. Phys. Act..

[3] Guthold R., Stevens G.A., Riley L.M., Bull F.C. (2020). Global Trends in Insufficient Physical Activity among Adolescents: A Pooled Analysis of 298 Population-Based Surveys with 1·6 Million Participants. Lancet Child Adolesc. Health.

[4] Ding D., Varela A.R., Bauman A.E., Ekelund U., Lee I.-M., Heath G., Katzmarzyk P.T., Reis R., Pratt M. (2020). Towards Better Evidence-Informed Global Action: Lessons Learnt from the Lancet Series and Recent Developments in Physical Activity and Public Health. Br. J. Sports Med..

[5] Messing S., Rütten A., Abu-Omar K., Ungerer-Röhrich U., Goodwin L., Burlacu I., Gediga G. (2019). How Can Physical Activity Be Promoted Among Children and Adolescents? A Systematic Review of Reviews Across Settings. Front. Public Health.

[6] Inchley J., Currie D., Young T., Samdal O., Torsheim T., Augustson L., Mathison F., Aleman-Diaz A., Molcho M., Weber M. (2016). Growing up Unequal: Gender and Socioeconomic Differences in Young People’s Health and Well-Being. Health Behaviour in School-Aged Children (HBSC) Study: International Report from the 2013/2014 Survey. Health Policy for Children and Adolescents.

[7] Strum R. (2005). Childhood Obesity—What We Can Learn from Existing Data on Societal Trends, Part 1. Prev. Chronic Dis..

[8] WHO (2015). Denmark Physical Activity Factsheet.

[9] Norris E., Shelton N., Dunsmuir S., Duke-Williams O., Stamatakis E. (2015). Physically Active Lessons as Physical Activity and Educational Interventions: A Systematic Review of Methods and Results. Prev. Med..

[10] Routen A.C., Chalkley A.E., Sherar L.B. (2017). Getting a GRIP (Getting Research into Practice) on Movement Integration in the School Classroom. Phys. Ther. Rev..

[11] Erwin H., Fedewa A., Beighle A., Ahn S. (2012). A Quantitative Review of Physical Activity, Health, and Learning Outcomes Associated with Classroom-Based Physical Activity Interventions. J. Appl. Sch. Psychol..

[12] Bentsen P., Bølling M., Mygind L., Schneller M.B., Stevenson M.P., Mygind E. (2019). Greening education: Outdoor learning in natural settings as an “add-in” holistic school-based health promotion approach for children and young people. Physical Activity in Natural Settings: Green Exercise and Blue Mind.

[13] Bentsen P., Mygind L., Elsborg P., Nielsen G., Mygind E. (2021). Education Outside the Classroom as Upstream School Health Promotion: ‘Adding-in’ Physical Activity into Children’s Everyday Life and Settings. Scand. J. Public Health.

[14] Ward J.S., Duncan J.S., Jarden A., Stewart T. (2016). The Impact of Children’s Exposure to Greenspace on Physical Activity, Cognitive Development, Emotional Wellbeing, and Ability to Appraise Risk. Health Place.

[15] Kuo M., Browning M.H.E.M., Sachdeva S., Lee K., Westphal L. (2018). Might School Performance Grow on Trees? Examining the Link Between “Greenness” and Academic Achievement in Urban, High-Poverty Schools. Front. Psychol..

[16] Hall N., McDonald G.K., Hay J., Defries D., Pryce R. (2016). Effect of Activity Type on Youth Physical Activity during Structured Activity Sessions. Health Behav. Policy Rev..

[17] Dyment J.E., Bell A.C., Lucas A.J. (2009). The Relationship between School Ground Design and Intensity of Physical Activity. Child. Geogr..

[18] Dyment J.E., Bell A.C. (2007). Active by Design: Promoting Physical Activity through School Ground Greening. Child. Geogr..

[19] van Dijk-Wesselius J.E., Maas J., Hovinga D., van Vugt M., van den Berg A.E. (2018). The Impact of Greening Schoolyards on the Appreciation, and Physical, Cognitive and Social-Emotional Well-Being of Schoolchildren: A Prospective Intervention Study. Landsc. Urban Plan..

[20] Beames S., Higgins P., Nicol R. (2012). Learning Outside the Classroom: Theory and Guidelines for Practice.

[21] Bentsen P., Mygind E., Randrup T.B. (2009). Towards an Understanding of Udeskole: Education Outside the Classroom in a Danish Context. Int. J. Prim. Elem. Early Years Educ..

[22] Gräfe R., Harring M., Sahrakhiz S., Witte M.D. (2018). Lernen Und Bildung in Der Draußenschule. Ein Unterrichtskonzept Zur Schulentwicklung Und Schulöffnung [Education and Learning in the Outdoor School. A Teaching Concept for Development of Schools]. Grundschulzeitschrift.

[23] Marchant E., Todd C., Cooksey R., Dredge S., Jones H., Reynolds D., Stratton G., Dwyer R., Lyons R., Brophy S. (2019). Curriculum-Based Outdoor Learning for Children Aged 9-11: A Qualitative Analysis of Pupils’ and Teachers’ Views. PLoS ONE.

[24] Fiskum T.A., Jacobsen K. (2013). Outdoor Education Gives Fewer Demands for Action Regulation and an Increased Variability of Affordances. J. Adventure Educ. Outdoor Learn..

[25] Mygind E. (2016). Physical Activity during Learning inside and Outside the Classroom. Health Behav. Policy Rev..

[26] Mygind E. (2007). A Comparison between Children’s Physical Activity Levels at School and Learning in an Outdoor Environment. J. Adventure Educ. Outdoor Learn..

[27] Schneller M.B., Duncan S., Schipperijn J., Nielsen G., Mygind E., Bentsen P. (2017). Are Children Participating in a Quasi-Experimental Education Outside the Classroom Intervention More Physically Active?. BMC Public Health.

[28] Quay J., Gray T., Thomas G., Allen-Craig S., Asfeldt M., Andkjaer S., Beames S., Cosgriff M., Dyment J., Higgins P. (2020). What Future/s for Outdoor and Environmental Education in a World That Has Contended with COVID-19?. J. Outdoor Environ. Educ..

[29] Becker C., Lauterbach G., Spengler S., Dettweiler U., Mess F. (2017). Effects of Regular Classes in Outdoor Education Settings: A Systematic Review on Students’ Learning, Social and Health Dimensions. Int. J. Environ. Res. Public Health.

[30] Schneller M.B., Schipperijn J., Nielsen G., Bentsen P. (2017). Children’s Physical Activity during a Segmented School Week: Results from a Quasi-Experimental Education outside the Classroom Intervention. Int. J. Behav. Nutr. Phys. Act..

[31] Grønningsæter I., Hallås O., Kristiansen T., Nævdal F. (2007). Fysisk Aktivitet Hos 11–12-Åringar i Skulen [Physical Activity in 11-12 Year Olds at School]. Tidsskr. Den. Nor. Legeforening.

[32] Bølling M., Otte C.R., Elsborg P., Nielsen G., Bentsen P. (2018). The Association between Education outside the Classroom and Students’ School Motivation: Results from a One-School-Year Quasi-Experiment. Int. J. Educ. Res..

[33] Bølling M., Niclasen J., Bentsen P., Nielsen G. (2019). Association of Education Outside the Classroom and Pupils’ Psychosocial Well-Being: Results from a School Year Implementation. J. Sch. Health.

[34] Otte C.R., Bølling M., Stevenson M.P., Nielsen G., Bentsen P., Ejbye-Ernst N. (2019). Education outside the Classroom Increases Children’s Reading Competencies: Results from a One-Year Danish Quasi-Experimental Study. Int. J. Educ. Res..

[35] Nielsen G., Mygind E., Bolling M., Otte C.R., Schneller M.B., Schipperijn J., Ejbye-Ernst N., Bentsen P. (2016). A Quasi-Experimental Cross-Disciplinary Evaluation of the Impacts of Education outside the Classroom on Pupils’ Physical Activity, Well-Being and Learning: The TEACHOUT Study Protocol. BMC Public Health.

[36] Barfod K., Ejbye-Ernst N., Mygind L., Bentsen P. (2016). Increased Provision of Udeskole in Danish Schools: An Updated National Population Survey. Urban For. Urban Green..

[37] Bentsen P., Schipperijn J., Jensen F.S. (2013). Green Space as Classroom: Outdoor School Teachers’ Use, Preferences and Ecostrategies. Landsc. Res..

[38] The Danish Ministry of Education (2014). Improving the Public School—Overview of Reform of Standards in the Danish Public School (Primary and Lower Secondary Education).

[39] The TEACHOUT Intervention TIDieR Checklist. Version 1.00.03. https://udeundervisning.dk/TEACHOUT/TEACHOUT_TIDieR.pdf.

[40] Schneller M.B., Bentsen P., Nielsen G., Brønd J.C., Ried-Larsen M., Mygind E., Schipperijn J. (2017). Measuring Children’s Physical Activity: Compliance Using Skin-Taped Accelerometers. Med. Sci. Sports Exerc..

[41] Duncan S., Stewart T., Mackay L., Neville J., Narayanan A., Walker C., Berry S., Morton S. (2018). Wear-time compliance with a dual-accelerometer system for capturing 24-hour behavioural profiles in children and adults. Int. J. Environ. Res. Public Health..

[42] Bates D., Maechler M., Bolker B., Walker S., Christensen R.H.B., Singmann H., Dai B., Scheipl F., Grothendieck G., Green P. (2020). Lme4: Linear Mixed-Effects Models Using “Eigen” and S4. https://cran.r-project.org/package=lme4.

[43] Kuznetsova A., Brockhoff P.B., Christensen R.H.B., Jensen S.P. (2017). LmerTest: Tests in Linear Mixed Effects Models. J. Stat. Softw..

[44] RStudio (2020). RStudio: Integrated Development for R. Version 1.2. 5042.

[45] Lenth R., Buerkner P., Herve M., Love J., Riebl H., Singmann H. (2020). Emmeans: Estimated Marginal Means, Aka Least-Squares Means. https://cran.r-project.org/package=emmeans.

[46] Becker C., Schmidt S., Neuberger E.W.I., Kirsch P., Simon P., Dettweiler U. (2019). Children’s Cortisol and Cell-Free DNA Trajectories in Relation to Sedentary Behavior and Physical Activity in School: A Pilot Study. Front. Public Health.

[47] Amagasa S., Machida M., Fukushima N., Kikuchi H., Takamiya T., Odagiri Y., Inoue S. (2018). Is Objectively Measured Light-Intensity Physical Activity Associated with Health Outcomes after Adjustment for Moderate-to-Vigorous Physical Activity in Adults? A Systematic Review. Int. J. Behav. Nutr. Phys. Act..

[48] Fuezeki E., Engeroff T., Banzer W. (2017). Health Benefits of Light-Intensity Physical Activity: A Systematic Review of Accelerometer Data of the National Health and Nutrition Examination Survey (NHANES). Sports Med..

[49] Rose G. (2001). Sick Individuals and Sick Populations. Int. J. Epidemiol..

[50] Waite S., Bølling M., Bentsen P. (2015). Comparing Apples and Pears?: A Conceptual Framework for Understanding Forms of Outdoor Learning through Comparison of English Forest Schools and Danish *Udeskole*. Environ. Educ. Res..

[51] Browning M.H.E.M., Rigolon A. (2019). School Green Space and Its Impact on Academic Performance: A Systematic Literature Review. Int. J. Environ. Res. Public Health.

[52] Mygind L., Kjeldsted E., Hartmeyer R., Mygind E., Bølling M., Bentsen P. (2019). Mental, Physical and Social Health Benefits of Immersive Nature-Experience for Children and Adolescents: A Systematic Review and Quality Assessment of the Evidence. Health Place.

[53] McCrorie P.R., Fenton C., Ellaway A. (2014). Combining GPS, GIS, and Accelerometry to Explore the Physical Activity and Environment Relationship in Children and Young People-a Review. Int. J. Behav. Nutr. Phys. Act..

[54] Bentsen P., Jensen F.S., Mygind E., Randrup T.B. (2010). The Extent and Dissemination of Udeskole in Danish Schools. Urban For. Urban Green..

[55] Atkin A.J., Sharp S.J., Harrison F., Brage S., Van Sluijs E.M. (2016). Seasonal Variation in Children’s Physical Activity and Sedentary Time. Med. Sci. Sports Exerc..

[56] Ryan R.M., Deci E.L. (2020). Intrinsic and Extrinsic Motivation from a Self-Determination Theory Perspective: Definitions, Theory, Practices, and Future Directions. Contemp. Educ. Psychol..

[57] Schochet P.Z. (2008). Guidelines for Multiple Testing in Impact Evaluations of Educational Interventions. Final Report.

[58] Melnick H., Darling-Hammond L. (2020). Reopening Schools in the Context of COVID-19: Health and Safety Guidelines from Other Countries. Policy Brief.

