# Supplementary material for: The Association between Education Outside the Classroom and Physical Activity: Differences Attributable to the Type of Space?
Source: Children (Basel). 2021 Jun 7;8(6):486. doi: 10.3390/children8060486 (PMC8227423; doi:10.3390/children8060486)
Supplement: Supplementary file 1 [file children-08-00486-s001.zip › children-1227476-supplementary.pdf]

Supplementary Materials, Table S1. Sample of device-measured PA cases and EOtC sessions.

School day settings		EOtC sessions					Pupils' descriptive			PA cases			
**Category**	**Researcher agreement of EOtC category**	**EOtC place data**	**Month**	**Session duration (transport and breaks incl.) (min)**	**Active (A) or passive (P) transport duration (min)**	**Break/free time duration (min)**	**Sex (% Girls)**	**Age[f] (y)**	**BMI**	***n* cases**	**SED (%)**	**LPA (%)**	**MVPA (%)**
Without PE or EOtC[a]	-	-	-	-	-	-	60.3%	10.82	17.66	1426	41.72	49.22	9.05
PE (without EOtC)[b]	-	-	-	-	-	-	58.5%	10.85	17.55	521	37.16	50.11	12.73
Green EOtC[c]	100%	Asserbo castle ruins and the surrounding forest.	April	300	60 (A)	0	35.3%	11.60	18.03	17	30.71	58.01	11.29
Green EOtC[c]	66%	Nature and green spaces. Outdoor. Forest in proximity to school.	February	210	15 (A)	15	50.0%	9.37	16.77	8	18.57	56.90	24.53
Green EOtC[c]	66%	Culture and society institutions. Outdoor. Assistens Cemetery in Copenhagen City.	January	165	0	50	50.0%	12.69	17.35	32	38.42	55.46	6.12
Green EOtC[c]	100%	Nature and green spaces. Outdoor. Kvark Mill outside the city of Vejle.	January	360	25 (A)	60	50.0%	10.50	16.37	14	29.42	57.54	13.04
Green EOtC[c]	100%	Nature and green spaces. Outdoor. Walk route around Arden city / Scout yard.	March	210	10 (A)	30	56.5%	9.75	17.33	23	40.45	50.06	9.49
Green EOtC[c]	100%	Nature and green spaces. Outdoor.	March	255	30 (A)	45	40.0%	10.60	16.06	15	29.50	57.38	13.13
Green EOtC[c]	100%	Nature and green spaces. Outdoor. Gurre castle ruins.	May	300	120 (A)	45	70.0%	10.41	17.81	20	36.96	51.98	11.05
Green EOtC[c]	66%	Natureskoler. Outdoor. The plant breeding station.	May	300	40 (A)	45	68.4%	10.38	17.68	19	36.56	52.42	11.02
Green EOtC[c]	100%	Nature and green spaces. Outdoor.	May	330	30 (A)	60	68.8%	9.53	16.97	16	24.58	62.76	12.66
Green EOtC[c]	100%	Nature and green spaces. Outdoor. Boserup Naturecenter.	November	370	100 (P)	30	57.1%	11.50	16.75	21	32.76	60.74	6.51
Non-green EOtC[d]	66%	Culture and society institutions. Out- and indoor. Ordrupgaard museum.	January	300	120 (P)	30	88.2%	10.69	16.36	17	43.97	51.92	4.11
Non-green EOtC[d]	100%	Culture and society institutions. Indoor. Aalborg Theater.	March	205	90 (P)	0	56.5%	9.75	17.33	23	35.56	54.26	10.18
Non-green EOtC[d]	100%	Virksomheder. Indoor. Jyllinge grocery stores.	November	90	10 (A)	0	63.2%	11.50	16.76	19	43.27	48.37	8.36
School-ground EOtC[e]	100%	Nature and green spaces. Outdoor. The school's outdoor areas and scrub.	April	255	10 (A)	30	76.0%	9.61	16.80	25	34.29	56.82	8.88
School-ground EOtC[e]	100%	The school's land. Outdoor. The football field.	December	90	0	10	45.5%	12.44	19.06	11	46.79	46.03	7.19
School-ground EOtC[e]	100%	The school's land. Out- and indoor.	January	165	15 (A)	10	43.5%	12.68	17.29	23	42.07	50.35	7.57
School-ground EOtC[e]	100%	The school's land. Out- and indoor. The local area of Torsted city.	March	640	5 (A)	30	57.1%	10.38	16.85	14	45.25	47.81	6.94

N=2264. min., Minutes. y, Years. All pupils' descriptive and PA case values are mean across school day setting. [a] Without PE or EOtC: School day without a PE lesson or an EOtC session. [b] PE (without EOtC): School day with a PE lesson and without an EOtC session. [c] Green EOtC: School day with an EOtC session primarily conducted in e.g. parks, forests and nature schools and without a PE lesson. [d] Non-green EOtC: School day with an EOtC

session primarily conducted in cultural and societal institutions or companies and without a PE lesson. [e] School-ground EOtC: School day with an EOtC session primarily conducted at the school-ground and without a PE lesson. [f] Age at 1st of January 2015.

Supplementary Materials, Table S2. SED full mixed model results.

Fixed effects	Estimate	Std. Error	t	*p*
(Intercept)	-7.196e+00	7.646e+00	-0.941	0.34952
Non-green EOtC	6.935e+00	2.477e+00	2.800	0.00519
School-ground EOtC	2.080e+00	2.073e+00	1.004	0.31588
PE (without EOtC)	2.195e-01	2.679e+00	0.082	0.93472
Without PE or EOtC	4.654e+00	2.651e+00	1.756	0.07944
BMI	3.726e-01	1.303e-01	2.860	0.00440
Age	3.075e+00	6.184e-01	4.972	9.75e-06
Sex (Girl = 1)	7.117e+00	7.111e-01	10.008	< 2e-16
EOtC session duration (transport and breaks incl.) (min.)	5.130e-03	7.462e-03	0.687	0.49198
EOtC active transport duration (min.)	-3.807e-02	2.908e-02	-1.309	0.19117
EOtC passive transport duration (min.)	-3.419e-02	2.116e-02	-1.616	0.10639
EOtC break/free time duration (min.)	-7.445e-02	5.290e-02	-1.407	0.16019

N=2,264. min., Minutes.

Supplementary Materials, Table S3. LPA full mixed model results.

Fixed effects	Estimate	Std. Error	t	*p*
(Intercept)	8.179e+01	6.217e+00	13.156	< 2e-16
Non-green EOtC	-6.083e+00	2.046e+00	-2.973	0.003002
School-ground EOtC	-5.868e-01	1.708e+00	-0.343	0.731346
PE (without EOtC)	-2.368e+00	2.209e+00	-1.072	0.284081
Without PE or EOtC	-3.316e+00	2.185e+00	-1.518	0.129493
BMI	-5.206e-01	1.057e-01	-4.927	1.1e-06
Age	-1.882e+00	5.021e-01	-3.749	0.000626
Sex (Girl = 1)	7.055e-01	5.770e-01	1.223	0.221973
EOtC session duration (transport and breaks incl.) (min.)	-8.292e-03	6.154e-03	-1.347	0.178238
EOtC active transport duration (min.)	2.581e-02	2.383e-02	1.083	0.279515
EOtC passive transport duration (min.)	5.806e-02	1.750e-02	3.317	0.000927
EOtC break/free time duration (min.)	1.123e-01	4.350e-02	2.582	0.010365

N=2,264. min., Minutes.

Supplementary Materials, Table S4. MVPA full mixed model results.

Fixed effects	Estimate	Std. Error	t	*p*
(Intercept)	2.547e+01	3.806e+00	6.693	5.89e-09
Non-green EOtC	-1.102e+00	1.030e+00	-1.070	0.284970
School-ground EOtC	-1.783e+00	8.826e-01	-2.020	0.043670
PE (without EOtC)	1.978e+00	1.131e+00	1.749	0.080524
Without PE or EOtC	-1.477e+00	1.119e+00	-1.319	0.187287
BMI	1.585e-01	6.369e-02	2.489	0.013106
Age	-1.198e+00	3.156e-01	-3.795	0.000465
Sex (Girl = 1)	-7.834e+00	3.459e-01	-22.646	< 2e-16
EOtC session duration (transport and breaks incl.) (min.)	4.625e-03	3.143e-03	1.472	0.141411
EOtC active transport duration (min.)	1.399e-02	1.289e-02	1.085	0.278182
EOtC passive transport duration (min.)	-2.521e-02	8.676e-03	-2.906	0.003708
EOtC break/free time duration (min.)	-5.151e-02	2.309e-02	-2.231	0.026023

N=2,264. min., Minutes.

Supplementary Materials, Table S5. Girls' SED full mixed model results.

Fixed effects	Estimate	Std. Error	t value	*p*
(Intercept)	-5.288e+00	8.972e+00	-0.589	0.5577
Non-green EOtC	6.967e+00	3.077e+00	2.264	0.0238
School-ground EOtC	2.481e+00	2.565e+00	0.967	0.3337
PE (without EOtC)	-2.080e+00	3.395e+00	-0.613	0.5403
Without PE or EOtC	3.364e+00	3.365e+00	1.000	0.3178
BMI	3.864e-01	1.530e-01	2.525	0.0121
Age	3.728e+00	7.226e-01	5.159	7.77e-06
EOtC session duration (transport and breaks incl.) (min.)	3.859e-03	9.506e-03	0.406	0.6849
EOtC active transport duration (min.)	-2.840e-02	3.410e-02	-0.833	0.4054
EOtC passive transport duration (min.)	-3.716e-02	2.604e-02	-1.427	0.1540
EOtC break/free time duration (min.)	-1.198e-01	6.598e-02	-1.815	0.0700

n=1,349. min., Minutes.

Supplementary Materials, Table S6. Girls' LPA full mixed model results.

Fixed effects	Estimate	Std. Error	t	*p*
(Intercept)	9.124e+01	7.217e+00	12.642	< 2e-16
Non-green EOtC	-7.261e+00	2.585e+00	-2.809	0.00507
School-ground EOtC	-1.049e+00	2.150e+00	-0.488	0.62583
PE (without EOtC)	-1.122e+00	2.839e+00	-0.395	0.69277
Without PE or EOtC	-3.404e+00	2.814e+00	-1.210	0.22675
BMI	-5.483e-01	1.296e-01	-4.231	3.08e-05
Age	-2.707e+00	5.730e-01	-4.723	5.95e-05
EOtC session duration (transport and breaks incl.) (min.)	-6.066e-03	7.953e-03	-0.763	0.44585
EOtC active transport duration (min.)	-4.256e-04	2.840e-02	-0.015	0.98805
EOtC passive transport duration (min.)	5.465e-02	2.195e-02	2.490	0.01294
EOtC break/free time duration (min.)	1.235e-01	5.501e-02	2.244	0.02521

n=1,349. min., Minutes.

Supplementary Materials, Table S7. Girls' MVPA full mixed model results.

Fixed effects	Estimate	Std. Error	t	*p*
(Intercept)	1.386e+01	3.815e+00	3.632	0.00057
Non-green EOtC	1.645e-01	1.055e+00	0.156	0.87613
School-ground EOtC	-1.655e+00	8.934e-01	-1.852	0.06428
PE (without EOtC)	3.064e+00	1.184e+00	2.588	0.00982
Without PE or EOtC	-9.761e-02	1.175e+00	-0.083	0.93379
BMI	1.652e-01	6.370e-02	2.594	0.00994
Age	-9.948e-01	3.192e-01	-3.117	0.00324
EOtC session duration (transport and breaks incl.) (min.)	3.587e-03	3.304e-03	1.085	0.27802
EOtC active transport duration (min.)	2.434e-02	1.241e-02	1.962	0.05031
EOtC passive transport duration (min.)	-1.908e-02	8.804e-03	-2.168	0.03042
EOtC break/free time duration (min.)	-1.585e-02	2.350e-02	-0.675	0.50016

n=1,349. min., Minutes.

Supplementary Materials, Table S8. Boys' SED full mixed model results.

Fixed effects	Estimate	Std. Error	t	*p*
(Intercept)	-6.598346	10.816201	-0.610	0.54455
Non-green EOtC	7.741242	4.132325	1.873	0.06154
School-ground EOtC	3.211576	3.331298	0.964	0.33586
PE (without EOtC)	4.580792	4.179014	1.096	0.27378
Without PE or EOtC	7.530674	4.116529	1.829	0.06822
BMI	0.318511	0.228659	1.393	0.16485
Age	2.782341	0.811750	3.428	0.00243
EOtC session duration (transport and breaks incl.) (min.)	0.006310	0.011351	0.556	0.57864
EOtC active transport duration (min.)	-0.035696	0.049717	-0.718	0.47328
EOtC passive transport duration (min.)	-0.022534	0.036535	-0.617	0.53759
EOtC break/free time duration (min.)	0.006447	0.081746	0.079	0.93727

n=915. min., Minutes.

Supplementary Materials, Table S9. Boys' LPA full mixed model results.

Fixed effects	Estimate	Std. Error	t	*p*
(Intercept)	72.889617	8.027624	9.080	7.37e-11
Non-green EOtC	-5.066048	3.280006	-1.545	0.1231
School-ground EOtC	-1.278260	2.605108	-0.491	0.6241
PE (without EOtC)	-5.555430	3.277139	-1.695	0.0913
Without PE or EOtC	-4.600466	3.227225	-1.426	0.1553
BMI	-0.406483	0.174623	-2.328	0.0207
Age	-1.089869	0.585138	-1.863	0.0832
EOtC session duration (transport and breaks incl.) (min.)	-0.010627	0.008913	-1.192	0.2342
EOtC active transport duration (min.)	0.050680	0.038786	1.307	0.1925
EOtC passive transport duration (min.)	0.061482	0.029224	2.104	0.0358
EOtC break/free time duration (min.)	0.069208	0.062762	1.103	0.2731

n=915. min., Minutes.

Supplementary Materials, Table S10. Boys' MVPA full mixed model results.

Fixed effects	Estimate	Std. Error	t	*p*
(Intercept)	31.856913	6.597056	4.829	1.31e-05
Non-green EOtC	-3.964393	2.091121	-1.896	0.0585
School-ground EOtC	-2.910674	1.784473	-1.631	0.1037
PE (without EOtC)	-0.341293	2.170430	-0.157	0.8751
Without PE or EOtC	-4.182508	2.140540	-1.954	0.0512
BMI	0.140237	0.118162	1.187	0.2364
Age	-1.490889	0.535594	-2.784	0.0097
EOtC session duration (transport and breaks incl.) (min.)	0.006821	0.005902	1.156	0.2484
EOtC active transport duration (min.)	-0.014147	0.026598	-0.532	0.5952
EOtC passive transport duration (min.)	-0.044758	0.017979	-2.490	0.0130
EOtC break/free time duration (min.)	-0.119845	0.046200	-2.594	0.0101

n=915. min., Minutes.
